# How Acute Postoperative Pain Impacts Patient Satisfaction After Lower Limb Fracture Surgery: A 14-Day Follow-Up Study

**DOI:** 10.7759/cureus.99055

**Published:** 2025-12-12

**Authors:** Ayisha Sultana, Shyam Sunder, Velavan Anandan, Prince Solomon, Akash Deep

**Affiliations:** 1 Orthopaedics and Trauma, Pondicherry Institute of Medical Sciences, Pondicherry, IND; 2 Community Medicine, Pondicherry Institute of Medical Sciences, Pondicherry, IND

**Keywords:** epidural anaesthesia, fracture fixation external, internal fixation, lower extremity trauma, orthopaedics surgery, patient satisfaction, postoperative pain

## Abstract

Background: Postoperative pain following lower limb fracture surgery is frequently severe, yet the correlation between this acute pain trajectory and patient satisfaction with care remains poorly understood. This study investigated whether effective early pain relief correlates directly with improved patient satisfaction within the first 14 postoperative days.

Methodology: A prospective, longitudinal observational study was conducted on 58 adult patients undergoing surgical fixation for lower limb fractures. Pain levels (visual analog scale, VAS) and patient satisfaction (five-point Likert scale) were assessed at two timepoints: 48 hours and 14 days post-surgery.

Results: Mean VAS scores demonstrated a significant reduction from 8.88 ± 0.87 at 48 hours to 4.28 ± 1.1 by day 14 (p value <0.001). Despite initial widespread dissatisfaction, with only one patient (1.7%) satisfied at 48 hours, overall patient satisfaction increased steeply to 52 patients (89.7%) by day 14. Univariate analysis revealed that the type of anesthesia, method of primary analgesia (epidural vs. intravenous), or demographic factors were not significant predictors of patient satisfaction, while the type of fracture fixation (internal vs. external) significantly affected the outcome (p value <0.05).

Conclusion: These findings indicate that pain reduction alone does not exclusively drive satisfaction. Instead, satisfaction appears to be heavily influenced by the type of fixation, effective perioperative communication, and the patient's psychological adaptation over time. However, the association found between fixation type and satisfaction could be highly confounded by underlying fracture severity. Prioritizing patient education may be more effective than focusing solely on absolute pain scores for enhancing the patient experience. The complexity and sequelae of the initial trauma may be the key drivers of long-term patient dissatisfaction rather than the choice of anesthesia/analgesia.

## Introduction

The experience of acute postoperative pain following fracture surgery is often perceived by patients as greater than anticipated. However, existing literature often fails to simultaneously explore this experience through the objective metric of pain severity and the subjective measure of patient satisfaction [1,2]. While a linear relationship between reduced pain and increased satisfaction is intuitively assumed, clinical practice demonstrates that patient satisfaction is a multifactorial construct. It is influenced by preoperative expectations, health literacy, and communication with the surgical team.

The distinction between the immediate (48 hours) and early recovery (two weeks) phases is critical, as patient priorities and levels of adaptation change during this period. At our institution, clinical observations suggested that patients managed with optimized regional anesthesia and multimodal pain regimens reported higher satisfaction, independent of standard confounding factors such as age or fixation method [3,4]. To rigorously examine this relationship, we prospectively measured pain trajectory and satisfaction in adult patients undergoing lower limb fracture fixation.

The objective of this study is to identify factors that impact patient satisfaction using the visual analog scale (VAS), a validated, subjective measure for acute and chronic pain [1], and Likert’s scale. Likert-type scales are frequently used in medical education and medical education research [5]. We hypothesized that the evolution of pain severity and patient satisfaction would be independent over the first 14 postoperative days and that the chosen anesthesia and analgesia strategy - rather than demographic or surgical variables - would be the dominant determinant of satisfaction outcomes.

## Materials and methods

Study population and setting

Between May 2025 and June 2025, a cohort of 58 consecutive adults (aged ≥18 years) presenting with isolated lower limb fractures (femoral neck, intertrochanteric, femoral shaft, tibial, or ankle fractures) requiring operative fixation were enrolled. The study was conducted at the Department of Orthopaedics in a tertiary care centre in South India, serving both urban and rural populations.

Inclusion criteria comprised of adult patients (age ≥18 years) with isolated lower limb fractures (both open and closed) requiring operative fixation, the ability to provide informed consent, and the capacity to use standardized scales for pain and satisfaction assessment. Exclusion criteria were polytrauma or multiorgan injuries, documented psychiatric illness, neurological impairment affecting pain perception, and refusal to participate. The sample size was estimated for comparing paired VAS pain scores at 48 hours and 14 days after surgery. A change of 1.5 points on the 0-10 VAS was taken as clinically important based on postoperative pain studies [6]. With a two-sided α of 0.05, 90% power, and an assumed standard deviation of 2.5 points for the within-patient difference, the required sample size was 30 patients. Our study included 58 participants; the sample size was adequate to detect a clinically meaningful change in pain.

Data collection protocol

Structured face-to-face interviews were conducted by the research team on postoperative days 2 and 14 in the patient's room or clinic setting. Pain severity was quantified using the VAS, a 10 cm horizontal line scored from 0 ("no pain") to 10 ("worst pain imaginable"). We utilized a five-point Likert scale to assess three domains: satisfaction with pain relief effectiveness, overall satisfaction with the surgical experience, and confidence in recovery trajectory. The scale anchors were defined as 1 = Very Dissatisfied, 2 = Dissatisfied, 3 = Neutral, 4 = Satisfied, and 5 = Very Satisfied. Data collected included comorbid illnesses, previous surgeries, mode of injury, fracture site and type, operative approach, fixation method (internal vs. external), type of anesthesia (general vs. regional), and primary pain relief strategy (intravenous (IV) infusion vs. epidural catheter). The Institutional Ethics Committee of Pondicherry Institute of Medical Sciences granted approval for this study. Written informed consent was obtained from all participants prior to enrolment.

Statistical approach

Descriptive statistics were calculated as frequencies, percentages, means, and standard deviations. The change in continuous pain scores between days 2 and 14 was assessed using a paired t-test. The association between categorical variables and satisfaction at day 14 was analyzed using the chi-square test or Fisher's exact test, where cell counts were small. Statistical significance was defined as a two-tailed test (p < 0.05).

## Results

Sample characteristics

The cohort comprised 58 patients (38 male, 20 female) with a mean age of 50.2 ± 14.8 years. The majority were semi-skilled (46.6%) or skilled labourers (25.9%). Road traffic accidents accounted for 87.9% of the injury mechanisms. See Table 1 for the baseline demographic characteristics.

**Table 1:** Baseline demographic characteristics of the study participants (N=58)

Demographic Variable	n (%)
Age (years)	
20–40	13 (22.4)
41–60	23 (39.6)
>60	22 (37.9)
Gender	
Male	38 (65.5)
Female	20 (34.5)
Occupational Status	
Unemployed	11 (19.0)
Professional	5 (8.6)
Skilled laborer	15 (25.9)
Semi-skilled worker	27 (46.6)
Educational Status	
No formal education	4 (6.9)
Primary school	17 (29.3)
Secondary school	16 (27.6)
High school or beyond	21 (36.2)
Monthly Household Income (Indian National Rupee)	
<10,000	21 (36.2)
10,001–20,000	23 (39.7)
20,001–30,000	12 (20.7)
>30,000	2 (3.4)

Clinical and operative characteristics

The majority of patients (72.4% underwent internal fixation. Regional anesthesia (spinal or epidural) was administered to 96.6% of the cohort. For primary pain management, 67.2% received epidural catheters, while 32.8% were managed with IV analgesia alone. See Table 2 for the clinical and operative characteristics.

**Table 2:** Clinical and operative characteristics (N=58)

Characteristic	n (%)
Presence of Comorbid Illness	
Yes	13 (22.4)
No	45 (77.6)
Prior Surgical History	
Yes	10 (17.2)
No	48 (82.8)
Road traffic accident or fall	51 (87.9)
Sports-related	3 (5.2)
Occupational	3 (5.2)
Other	1 (1.7)
Type of Fixation Used	
Internal (plates, screws, nailing)	42 (72.4)
External	16 (27.6)
Anesthesia Type	
Regional (spinal/epidural)	56 (96.6)
General	2 (3.4)
Postoperative Analgesia Method	
Epidural catheter	39 (67.2)
Intravenous analgesia	19 (32.8)
Mobilization Status at Day 2	
Immobilized	10 (17.2)
Partial weight bearing	43 (74.1)
Full weight bearing	4 (6.9)
Non-weight bearing	1 (1.7)

Pain severity trajectory

**Figure 1:**
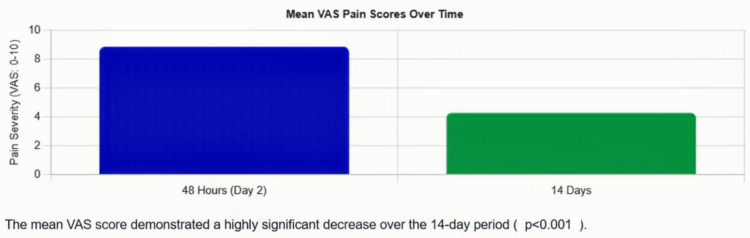
Mean visual analog scale (VAS) pain scores at 48 hours and 14 days The chart shows a clear decline in pain from 8.88/10 at 48 hours to 4.28/10 at 14 days, with error bars representing standard deviation. The trajectory illustrates the typical postoperative pain resolution pattern (p value <0.001)

On postoperative day two, the mean VAS was 8.88 ± 0.87. By day 14, the mean VAS had decreased substantially to 4.28 ± 1.12, representing a reduction of 4.60 points. This reduction was statistically highly significant (p < 0.001, paired t-test). See Figure 1 for the mean VAS pain scores at 48 hours and 14 days, and Table 3 for the VAS pain score at 48 hours and 14 days.

**Table 3:** Visual analog scale (VAS) pain score at 48 hours and 14 days This reduction in VAS score was statistically highly significant (p<0.001, paired t-test).

Outcome	Day 2	Day 14	p-value
Visual Analog Scale (VAS) Pain Score			
Mean ± SD	8.88 ± 0.87	4.28 ± 1.12	p < 0.001
Range	6–10	2–6	

Patient satisfaction over time

**Figure 2:**
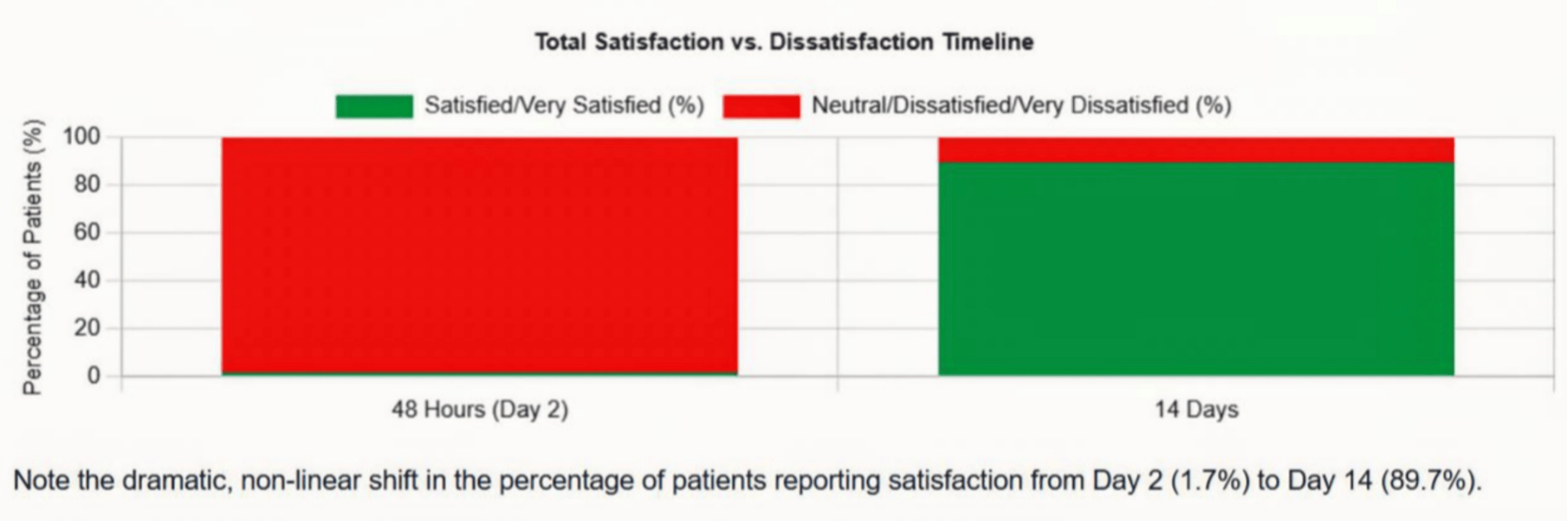
Distribution of patient satisfaction ratings at 48 hours vs. 14 days A stacked bar chart illustrates the dramatic shift in satisfaction: at 48 hours, approximately 82.8% express dissatisfaction, with only 1.7% satisfied. By day 14, the pattern inverts, with 89.7% satisfied and dissatisfaction dropping to 1.7%.

At 48 hours, only one patient (1.7%) reported satisfaction with pain relief, with 82.8% reporting dissatisfaction. The overall satisfaction with the surgical experience was similarly low. At 14 days, a near-complete reversal was observed, with 52 patients (89.7%) reporting satisfaction or marked satisfaction with pain relief and overall surgical experience. See Figure 2 for the distribution of patient satisfaction ratings at 48 hours vs. 14 days, and Table 4 for the patient satisfaction at 48 hours and 14 days.

**Table 4:** Patient satisfaction ratings at 48 hours and 14 days

Outcome	Day 2	Day 14
Patient Satisfaction Rating		
Very satisfied	0 (0%)	19 (32.8%)
Satisfied	1 (1.7%)	33 (56.9%)
Neutral	9 (15.5%)	5 (8.6%)
Dissatisfied	28 (48.3%)	1 (1.7%)
Very dissatisfied	20 (34.5%)	0 (0%)
Combined Satisfied/Very Satisfied	1 (1.7%)	52 (89.7%)

Predictors of patient satisfaction at day 14

Patients who received regional anesthesia (n=56) reported similar satisfaction rates compared to those receiving general anesthesia (n=2) (p value=0.198). However, this finding must be interpreted with extreme caution due to the very small sample size (n=2) in the general anesthesia group.

The type of analgesia also showed no significant statistical association with patient satisfaction (p=0.653). Meanwhile, both methods resulted in high overall satisfaction - 87.2% for epidural analgesia (n=34) and 94.7% for IV analgesia (n=18). This confirms that the choice of primary analgesic modality is not a strong predictor of the final satisfaction outcome. The observed trend of higher satisfaction in the IV group is noteworthy, potentially reflecting the avoidance of catheter-related complications sometimes associated with epidural use. Demographic and other clinical factors, such as age, gender, income, and education level, were not found to be statistically significant predictors of satisfaction at day 14. There was a significant statistical association between patient satisfaction and the type of fixation. Univariate analysis showed a significant satisfaction in patients who underwent internal fixation compared to the external fixation group (p value=0.043, statistically significant). See Table 5 for the univariate predictors of satisfaction.

**Table 5:** Univariate predictors of patient satisfaction at day 14 *Statistically significant (p<0.05). The association between categorical variables and satisfaction at day 14 was analysed using Fisher's exact test.

Variable	Satisfied/Very Satisfied	Neutral/Dissatisfied	p-value
Type of Anesthesia			
Regional	51 (91.1%)	5 (8.9%)	0.198
General	1 (50.0%)	1 (50.0%)	
Type of Analgesia			
Epidural	34 (87.2%)	5 (12.8%)	0.653
Intravenous	18 (94.7%)	1 (5.3%)	
Type of Fixation			
Internal	40 (95.2%)	2 (4.8%)	0.043*
External	12 (75.0%)	4 (25.0%)	
Gender			
Male	33 (86.8%)	5 (13.2%)	0.653
Female	19 (95.0%)	1 (5.0%)	
Age Group			
20–40 years	11 (78.6%)	3 (21.4%)	0.067
41–60 years	19 (90.5%)	2 (9.5%)	
>60 years	22 (95.7%)	1 (4.3%)	

Additionally, the calculation of the correlation coefficient between the VAS score (at 48 hours and 14 days ) and patient satisfaction score was done. The results showed a negative correlation between the VAS score and patient satisfaction score. See Table 6 for the correlation between VAS score and patient satisfaction score.

**Table 6:** Correlation between the VAS scores (at 48 hours, 14 days) and patient satisfaction scale There is a negative correlation between the VAS score and patient satisfaction score at 48 hours and 14 days.

Parameter	VAS (48 hours)	VAS (14 days)
Patient Satisfaction Score	-0.132	-0.125
	r=-0.132	r=-0.125

## Discussion

Pain is a highly subjective entity. Postoperative pain is acute pain due to surgical trauma with an inflammatory response and initiation of an afferent neuronal barrage that results in several unpleasant sensory, emotional, and psychological burdens. Patient satisfaction is a subjective measurement influenced by many variables and may be difficult to interpret.

The pain-satisfaction disparity

Our results underscore a significant disparity: severe pain at 48 hours did not predict low satisfaction at day 14. Furthermore, the 52% reduction in pain scores was disproportionate to the 88%-point surge in satisfaction. This phenomenon strongly suggests that satisfaction is not merely a consequence of pain relief but is heavily mediated by psychological adaptation, the achievement of early functional milestones, and the clarity of patient-provider communication. The initial high pain is an expected component of orthopaedic trauma recovery; however, the subsequent recovery trajectory allows non-pharmacological factors to influence the overall experience. Literature reviews shows strong association between patient satisfaction and postoperative pain scores [7], and optimizing postoperative pain management is a potential opportunity to satisfy the patient.

The role of anesthesia and analgesia

Literature evidence suggests that regional anesthesia was significantly associated with higher satisfaction as per established anesthetic guidelines. Regional techniques offer advantages such as reduced postoperative nausea/vomiting [8,9], faster cognitive recovery, and a superior patient-perceived pain control profile [10]. However, in our study, no significant association was found (p=0.198). This must be interpreted cautiously due to the minimal number of patients who received general anesthesia (n=2).

The statistically insignificant difference observed in satisfaction based on the analgesia method (p=0.653) suggests a delicate balance in pain management strategy. While epidural analgesia often provides superior numeric pain control, its use carries inherent risks of complications (e.g., urinary retention). Our data suggest that, for some patients, the avoidance of these complications via a well-managed IV analgesic regimen may translate into comparable or even higher satisfaction (as seen in the 94.7% satisfaction rate), despite potentially less aggressive numeric pain control. This emphasizes that patient comfort and complication avoidance are critical drivers of satisfaction, potentially outweighing the benefit of absolute zero pain.

Importance of expectation management and communication

Qualitative synthesis of patient remarks highlighted that initial dissatisfaction (day two) was often rooted in uncertainty - specifically a lack of clarity regarding expected pain severity and the recovery timeline. Studies show that pain sensations are enhanced by negative emotions [11,12]. The dramatic improvement in satisfaction by day 14 is likely a function of patients receiving repeated information, their expectations normalizing, and the commencement of tangible functional gains (e.g., initial mobilization). This supports the growing body of literature that emphasizes the crucial role of information provision, care team responsiveness, and the congruence between expected and experienced reality in determining patient contentment. Future interventions should therefore focus on standardized, timely patient education rather than relying solely on pharmacological interventions.

Demography, socioeconomic status, and satisfaction

The lack of association between satisfaction and demographic factors (age, income, education) is a powerful finding. Literature evidence shows varying results in relation to demographic parameters, as few studies showed that older age groups show higher satisfaction rates than younger age groups [13-15], whereas few studies show no significant association between age and patient satisfaction [7]. This indicates that, in our setting, where standardized surgical and pain management protocols were applied, the quality of care transcended socioeconomic stratification. This reinforces the principle that excellent clinical care, delivered with effective communication, is the universal driver of positive patient experience.

Type of fixation and satisfaction

The only significant factor having a statistically significant association with patient satisfaction was the type of fixation. Forty out of 42 patients who underwent internal fixation (n=40, 95.2%) reported better satisfaction rates than those who underwent external fixation (12 out of 16, 75%). Even though this is a statistically significant association, this needs to be analyzed with caution, as the patients who underwent external fixation were having open fractures, which inherently can cause more dissatisfaction because of the need for multiple surgeries, regular dressings, and the presence of heavy, not-so-aesthetic implants visible outside their bodies. Traditionally, too many external fixators are considered cumbersome by patients because of potential discomfort, restricted mobility, and prolonged care of pins, even though they are an excellent tool in the setting of open fracture treatment [16,17].

Study limitations

The study's findings are subject to several limitations, including the single-center design (limiting generalizability), a small sample size (n=58), which limits statistical power for complex subgroup analysis, and the reliance on subjective scales, which are prone to recall and response biases. Critically, the analysis of the type of fixation is severely limited by the small sample size in the external fixation group (16 in external fixation compared to 42 in internal fixation) and variability in the fracture prolife. Open fractures, which were treated by external fixation, are inherently a cause for poor satisfaction because of other factors discussed above. Furthermore, the brief 14-day follow-up period does not capture long-term satisfaction associated with functional recovery milestones.

## Conclusions

Our study failed to find an association between anesthesia/analgesia and patient satisfaction, likely due to underpowering/selection bias. The association between the type of fixation and patient satisfaction, even though statistically significant, needs detailed analysis to remove the confounding effect because of underlying fracture severity. This suggests that the complexity and sequelae of the initial trauma are key drivers of long-term patient dissatisfaction, not the choice of anesthesia/analgesia. Moreover, perioperative patient communication, effective expectation management, and optimizing regional pain control protocols are the principal drivers of a positive patient experience. Orthopaedic units should focus on these structural elements to enhance adherence to rehabilitation, improve functional outcomes, and build community trust.
